# Cerebellar Repetitive Transcranial Magnetic Stimulation (rTMS) With Modified Proprioceptive Neuromuscular Facilitation (PNF) Balloon Dilation for Dysphagia After Brainstem and Cerebellar Infarction: A Case Report

**DOI:** 10.7759/cureus.109417

**Published:** 2026-05-22

**Authors:** Li Gan, Longting Hu, Zhiping Zeng, Hongmei Lü, FengHao Yang, Wei Wang, YiYan Zou, PengXu Wei, Quan Wei

**Affiliations:** 1 Department of Rehabilitation Therapy, Affiliated Sichuan Provincial Rehabilitation Hospital of Chengdu University of Traditional Chinese Medicine, Chengdu, CHN; 2 Department of Neurological Rehabilitation, Affiliated Sichuan Provincial Rehabilitation Hospital of Chengdu University of Traditional Chinese Medicine, Chengdu, CHN; 3 Department of Radiology, Affiliated Sichuan Provincial Rehabilitation Hospital of Chengdu University of Traditional Chinese Medicine, Chengdu, CHN; 4 Department of Neurosurgery, Affiliated Sichuan Provincial Rehabilitation Hospital of Chengdu University of Traditional Chinese Medicine, Chengdu, CHN; 5 Department of Occupational Therapy, Affiliated Sichuan Provincial Rehabilitation Hospital of Chengdu University of Traditional Chinese Medicine, Chengdu, CHN; 6 Department of Rehabilitation Medicine, Third Hospital of Mianyang, Mianyang, CHN; 7 Department of Physical Therapy, Affiliated Sichuan Provincial Rehabilitation Hospital of Chengdu University of Traditional Chinese Medicine, Chengdu, CHN; 8 Department of Physical Therapy and Rehabilitation Medicine, National Research Center for Rehabilitation Technical Aids, Beijing Key Laboratory of Rehabilitation Technical Aids for Old-Age Disability, Beijing, CHN; 9 Department of Rehabilitation Medicine, Rehabilitation Medicine Center and Institute of Rehabilitation Medicine, West China Hospital, Sichuan University, Chengdu, CHN

**Keywords:** brainstem infarction, dysphagia, pnf-guided balloon dilation therapy, rehabilitation, rtms

## Abstract

Dysphagia resulting from bilateral cerebellar and brainstem infarction represents one of the most severe and challenging subtypes of post-stroke dysphagia in clinical practice. This study reports a case involving the application of cerebellar repetitive transcranial magnetic stimulation (rTMS) combined with modified proprioceptive neuromuscular facilitation (PNF)-guided balloon dilation therapy for dysphagia in a patient with bilateral cerebellar and brainstem infarction. The patient resumed full oral intake for all three daily meals and was able to drink fluids independently. A three-month follow-up confirmed sustained normal swallowing function with no signs of recurrence. The innovative "central-peripheral" combined therapeutic approach proposed in this study may represent a promising therapeutic option for the management of severe dysphagia following infratentorial stroke.

This study aimed to detail the swallowing function recovery process in a patient with severe post-stroke dysphagia (PSD) resulting from bilateral posterior cerebellar lobe infarction combined with brainstem infarction, following comprehensive intervention integrating cerebellar rTMS (central neuromodulation) and modified PNF-synchronized catheter balloon dilation (peripheral functional remodeling). Through this case, we seek to explore the feasibility, safety, and potential therapeutic efficacy of this combined regimen for such complex PSD. It is intended to provide novel clinical insights and practical evidence for developing individualized and precise swallowing rehabilitation strategies in the future.

## Introduction

Stroke currently represents the leading cause of the global burden of neurological diseases, with over 13 million new cases reported annually [1]. Post-stroke dysphagia (PSD) is a common complication, closely associated with increased mortality, reduced quality of life, and heightened healthcare economic burden in patients [2]. In patients with brainstem lesions, the incidence of PSD can reach as high as 80% [3]. It significantly increases the risk of aspiration pneumonia, malnutrition, dehydration, and mortality, while also prolonging hospital stays and exacerbating the healthcare burden. The dorsal swallowing group (DSG) and the ventral swallowing group (VSG) in the medulla oblongata together constitute the central pattern generator (CPG) for swallowing [4]. This CPG is responsible for integrating swallowing information, initiating and orchestrating the motor sequence of swallowing, and coordinating reflexive swallowing. Therefore, a brainstem stroke can lead to more severe dysphagia. Patients often present with significantly delayed initiation of the pharyngeal phase, insufficient laryngeal elevation, severe coordination disorders (timing disturbances) across all phases of swallowing, and incomplete opening or achalasia of the cricopharyngeus muscle [5]. Conventional single-modality rehabilitation approaches frequently yield limited therapeutic efficacy.

Repetitive transcranial magnetic stimulation (rTMS), as a non-invasive brain stimulation technique, has shown considerable potential for the neuromodulatory treatment of PSD by regulating the excitability of cortical and subcortical networks [6-8]. In recent years, cerebellar rTMS has emerged as a novel intervention strategy due to its ability to indirectly modulate the brainstem swallowing center and the contralateral cerebral motor cortex [9-11]. Among various peripheral intervention techniques, catheter balloon dilation is an effective physical method for treating cricopharyngeal achalasia [3,12]. The modified proprioceptive neuromuscular facilitation (PNF) technique synchronously combined with catheter balloon dilation therapy adopted in this study represents a significant technological integration and innovation [13-15].

## Case presentation

A 56-year-old male presented with a one-month history of dysphagia, coughing during swallowing, and unclear speech. He was conscious upon admission and had been relying on long-term nasogastric tube feeding. One month prior to admission (July 24, 2025), the patient experienced generalized weakness and dysphagia without an obvious precipitating factor, with no impairment of consciousness or motor function. He was subsequently evaluated and treated at an external hospital, where diagnoses of acute cerebral infarction and bacterial pneumonia were considered. Treatment included neurotrophic therapy, agents to improve circulation, and antiplatelet aggregation, after which his condition stabilized, and he was discharged, though dysphagia persisted. Current examination revealed restricted mandibular opening/closing, limited tongue movement (protrusion, lateralization, circumduction), impaired chewing motions, with reduced resistance against force, and hoarseness.

Cranial MRI demonstrated multiple cerebral infarctions involving the bilateral cerebellar hemispheres and medulla oblongata, bilateral basal ganglia, corpus callosum, bilateral centrum semiovale, and lateral ventricles, along with mild white matter demyelination. Diffusion-weighted imaging (DWI) revealed a large area of abnormal signal in the bilateral posterior cerebellar lobes, indicative of acute cerebral infarction. Additionally, punctate abnormal signals were present in the right ventral pons and right lateral medulla, suggestive of acute lacunar infarction (Figure 1, panels a-d).

**Figure 1:**
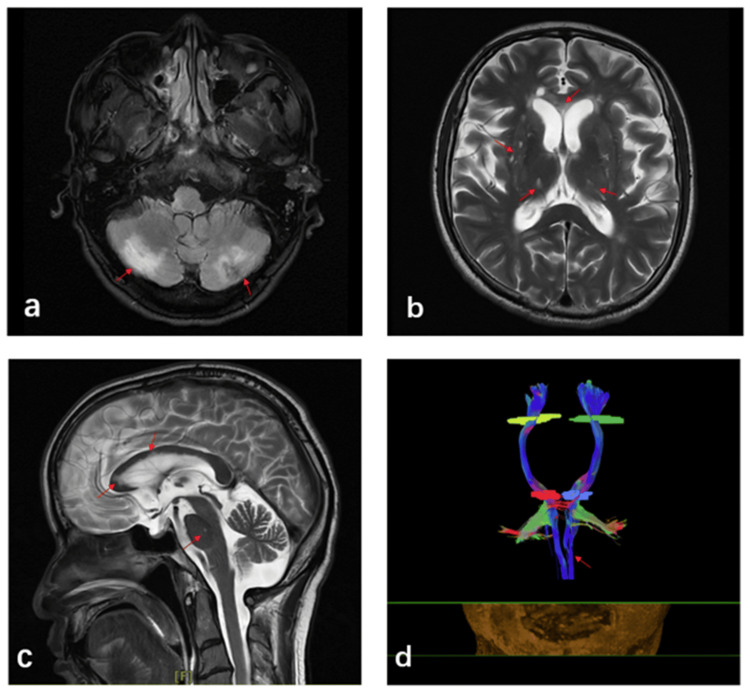
Cranial magnetic resonance imaging (MRI). (a) FLAIR imaging of bilateral cerebellar infarction (arrows), (b) multiple infarcts (arrows) in bilateral basal ganglia, thalamus, and corpus callosum, (c) sagittal: pontine and callosal infarcts (arrows), (d) diffusion tensor imaging (DTI) sequence of a medullary infarction (arrows).

Based on the patient's clinical presentation, a comprehensive clinical assessment of swallowing function was performed. The evaluation revealed that the laryngeal elevation amplitude was less than 2 cm, the Repetitive Saliva Swallowing Test result was 0 times/30 s, the Functional Oral Intake Scale score was level 1, and the Kubota water-swallowing test score was grade V. Videofluoroscopic swallowing study (VFSS) findings of pre-treatment were as follows: the patient exhibited significant difficulty initiating swallowing, markedly insufficient laryngeal elevation and anterior movement, and delayed swallowing initiation (Figure 2, panel a). Substantial barium residue was observed in the valleculae and pyriform sinuses. Even after multiple attempts with head turn and chin tuck maneuvers, only minimal residue clearance was achieved, accompanied by significant oral reflux. Barium spillage from the pyriform sinuses into the laryngeal vestibule was noted, leading to significant leakage and subsequent entry into the trachea, causing evident aspiration. Coughing efforts were observed but were weak and ineffective. Only approximately 1/10 of the bolus successfully entered the esophagus. Throughout the swallowing process, incomplete opening of the upper esophageal sphincter (cricopharyngeus muscle) was observed (Figure 3). Post-treatment (20 days) imaging showed minimal residue in the pyriform sinuses and valleculae, which was largely cleared by repeated swallows and cough reflex (Figure 2, panel b). The opening of the cricopharyngeus muscle remained incomplete.

**Figure 2:**
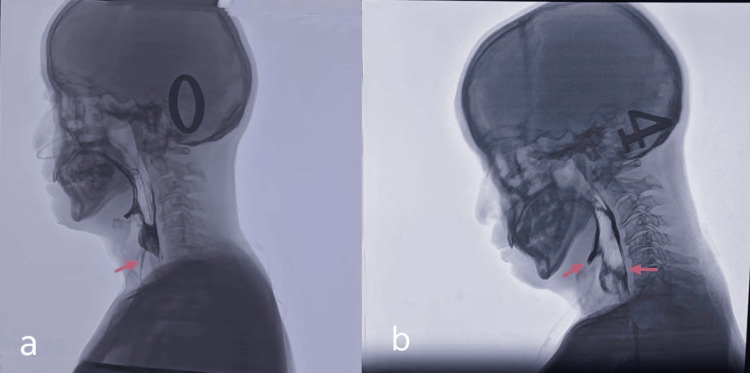
Videofluoroscopic swallowing study (VFSS) (arrows). (a) Pre-treatment and (b) post-treatment imaging.

**Figure 3:**
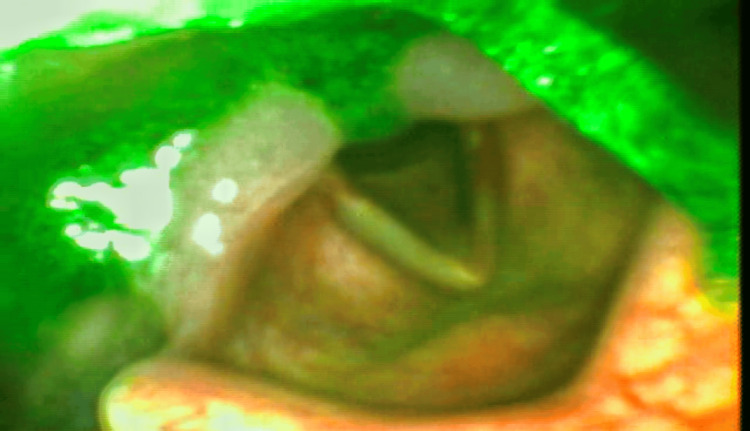
Aspiration and penetration observed during fiberoptic endoscopic evaluation of swallowing (FEES).

Following a comprehensive assessment of the patient's condition by our multidisciplinary team (MDT) and a detailed explanation of the swallowing treatment plan, informed consent was obtained from the patient. Pharmacological management primarily focused on secondary stroke prevention and neurotrophic medications. The rTMS intervention was administered by a physician from the neurological rehabilitation department using a MagTD magnetic field stimulator (Wuhan, China: Yiruide Medical). The bilateral stimulation targets were set 1 cm below the inion and 3 cm lateral to the midline bilaterally [11]. Stimulation parameters were set at a frequency of 10 Hz, with an intensity of 80% of the resting motor threshold (RMT). The protocol consisted of 80 trains, each comprising 10 pulses, with a 1-s stimulation duration and a 9-s inter-train interval. This resulted in a total of 800 pulses per stimulation site per session, with each session lasting approximately 13 min. Stimulation was administered five times per week, for a total of 12 sessions.

Modified PNF-guided balloon dilation therapy was performed within the 30-min therapeutic window following each rTMS session [16]. The specific procedural steps were as follows: prior to and during the modified balloon dilation procedure, PNF therapeutic patterns were applied to the head and neck region, mandible, and tongue. These included techniques such as dynamic reversals of antagonists, rhythmic stabilization, and hold-relax. Head/neck PNF diagonal pattern training was conducted (e.g., head flexion-rotation coupled with shoulder synergistic movements), along with head movements tracing a horizontal "8" pattern. Mandible resistance training for jaw opening/closing and PNF stretching for lateral mandibular shifts were performed. Lips/tongue exercises included lip-muscle resistance training using objects (e.g., a tongue depressor) and active tongue protrusion/retraction or cheek-pushing exercises, combined with the PNF "hold-relax" technique. Immediately preceding the dilation phase, preparatory maneuvers were conducted as follows: the patient's head was sequentially moved to the following positions, holding each for 8 s - right 45° up → right 45° down → neutral position → left 45° up → left 45° down → neutral position. This sequence was repeated for three to five sets per session. The overall treatment process is shown in Figure 4, panels a-d, and detailed technical procedures can be found in the appendix.

**Figure 4:**
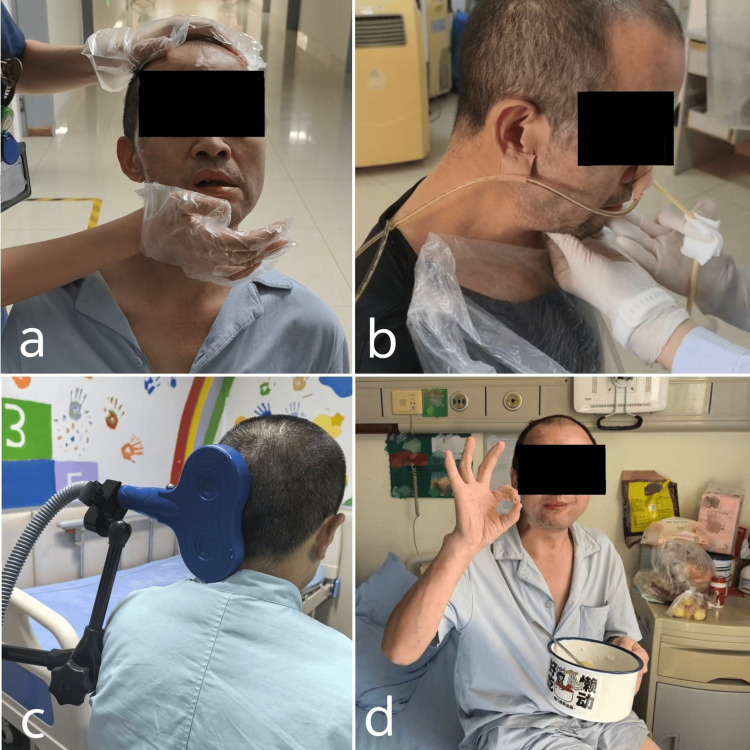
Overall treatment process. a: mandibular PNF therapy; b: modified PNF-guided balloon dilation therapy; c: cerebellar repetitive transcranial magnetic stimulation (rTMS); d: achievement of full oral diet prior to discharge.

Upon completion of 10 sessions, the Functional Oral Intake Scale (FOIS) score had increased to level 4, and the water swallowing test improved to grade III (Table 1). The patient achieved full oral intake of foods such as stewed pumpkin, steamed egg custard, and mashed potatoes, with a notable decrease in frothy oral secretions. After all 12 treatment sessions, the FOIS score reached level 6, and the water swallowing test improved to grade II. The patient resumed full oral intake for all three daily meals and fluids. A follow-up videofluoroscopic swallowing study conducted with 5-10 mL boluses of 60% w/v barium suspension, thin liquids, thick liquids, puree, and solid food revealed only minimal residue in the valleculae and pyriform sinuses. This residue was largely cleared with repeated swallowing efforts or cough, and oral reflux was absent. The penetration-aspiration scale (PAS) score decreased to level 1 [17]. The treatment successfully restored the patient's swallowing function, with concomitant significant improvement in hoarseness and speech articulation. Swallowing therapy was therefore concluded successfully, and the patient was discharged on a regular diet.

**Table 1:** Pre- and post-treatment swallowing assessment scores. FOIS: Functional Oral Intake Scale (scores range from 1 {nil by mouth} to 7 {total oral diet without restriction}); modified water swallow test: scores range from 1 (normal, swallows 30 mL water in <5 s without cough) to 5 (frequent coughing or unable to swallow); PAS: penetration-aspiration scale (scores range from 1 {no penetration} to 8 {silent aspiration}); VDS: videofluoroscopic dysphagia scale (scores range from 0 to 100; higher scores indicate more severe dysphagia); SSA: standardized swallowing assessment (scores range from 18 to 46; higher scores indicate more abnormal swallowing); GUSS: Gugging Swallowing Screen (scores range from 0 to 20; lower scores indicate higher aspiration risk)

Assessment (scale range)	Pre‑treatment score	Post‑treatment score (after 12 sessions)
FOIS (1-7)	1	6
Modified water swallow test (I-V)	V	II
PAS (1-8)	7	1
VDS (0-100)	90	23.5
SSA (18-46)	34	25
GUSS (0-20)	5	16

The entire treatment cycle lasted 20 days, comprising 12 combined therapy sessions. No adverse events were reported during this period. A telephone follow-up at three months post-treatment indicated sustained normal swallowing function with no signs of recurrence.

## Discussion

This study details and analyzes the complete clinical course of a patient with severe, refractory dysphagia resulting from bilateral posterior cerebellar lobe infarction combined with brainstem infarction, who achieved significant functional improvement following a comprehensive rehabilitation intervention centered on cerebellar rTMS combined with modified PNF-synchronized catheter balloon dilation. This outcome raises the possibility of an innovative treatment paradigm with potential synergistic effects for the clinical management of such complex central dysphagia.

The combined application of these two interventions may suggest synergistic effects that extend beyond simple addition. Specifically, the following speculative mechanisms are proposed, although they await verification in controlled studies: temporal window coupling - cerebellar rTMS is hypothesized to optimize the excitability and coordination of the central network from an “upstream” level, potentially improving the efficiency and timing of swallow command generation. This may create a more favorable “central preparedness state” for subsequent high‑intensity, high-precision peripheral training involving modified PNF‑balloon dilation. In other words, following neuromodulation with bilateral cerebellar rTMS, the neural circuits might be more effective at processing and responding to the complex sensory input provided by PNF and at executing the required active motor patterns. Bidirectional promotion of neuroplasticity may occur [18]. It has been speculated that rTMS-induced changes in cortical and subcortical excitability extend the time window for synaptic plasticity (e.g., long-term potentiation {LTP}) [11]. During this window, the intense sensorimotor feedback generated by synchronized, behaviorally relevant PNF-balloon dilation training could be more effectively encoded and consolidated within neural circuits, thereby accelerating the relearning and solidification of functional swallowing patterns. This notion aligns with the general principles of “time‑dependent” and “task‑specific” neuroplasticity, but direct evidence from the present case is lacking. The simultaneous addressing of structure and function through the combined protocol may concurrently target impairments at different levels, with rTMS addressing central coordination deficits (timing disorders and initiation delay), while modified balloon dilation directly targets peripheral structural and functional obstruction (incomplete cricopharyngeus opening), while also training muscle strength and coordination. This “multi‑point synchronous intervention” strategy could be relevant for complex post‑stroke dysphagia involving multifocal and multi‑level lesions, but this remains speculative.

As a single-case report, this study has inherent limitations as it cannot establish causality, and the observed outcomes may reflect spontaneous neurological recovery, concurrent conventional therapies, or placebo effects. The optimal treatment parameters, including rTMS frequency, intensity, total pulses, specific PNF techniques, and balloon dilation pressure/frequency, require determination through rigorous controlled studies. The hypotheses generated here should be tested in future trials with larger sample sizes, control groups, and blinded outcome assessments.

## Conclusions

In summary, this single-case observation provides preliminary, hypothesis‑generating insights into the clinical feasibility of a comprehensive treatment regimen combining cerebellar rTMS with modified PNF‑synchronized balloon dilation for managing complex dysphagia following brainstem and cerebellar infarction. It remains unclear whether the observed improvement reflects the combined intervention, spontaneous recovery, possible placebo effects, or concurrent conventional rehabilitation. Therefore, these findings should not be interpreted as evidence of therapeutic efficacy or clinical applicability. Instead, this report offers an exploratory reference protocol designed to inform future hypothesis‑driven research, including controlled trials, to rigorously evaluate the potential role of integrating central neuromodulation with patterned peripheral functional remodeling in facilitating swallowing network reorganization and behavioral relearning.

## Appendices

**Table 2:** PNF exercises for key orofacial muscle groups. PNF: proprioceptive neuromuscular facilitation

Muscle group	Movement	Purpose	PNF technique
Lingual muscles	Upward (toward the nasal tip), downward (toward the mandible), upper-left (45° angle between the nasal tip and the left mouth corner), lower-right (45° angle between the right mouth corner and the mandible), upper-right (45° angle between the nasal tip and the right mouth corner), lower-left (45° angle between the left mouth corner and the mandible)	Increase range of motion and modulate abnormal muscle tone. Enhance strength, improve range of motion, and improve stability	Rhythmic initiation, dynamic reversal, contract-relax, rhythmic initiation, stability reversal, dynamic reversal
1. Anterior belly of digastric, mylohyoid, and geniohyoid muscles	Jaw elevation	Increase range of motion	
	Jaw depression	Improve stability	
2. Lateral pterygoid muscle	Anterior-posterior	Enhance strength	
3. Ipsilateral medial pterygoid and contralateral lateral pterygoid muscles	Left-posterior	Enhance strength	
1+2+3	Left-anterior-superior to right-posterior-inferior, right-anterior-superior to left-posterior-inferior	Enhance strength and stability	

**Table 3:** PNF exercises for key craniocervical muscle groups. PNF: proprioceptive neuromuscular facilitation

Muscle group	Movement	Purpose	PNF technique
Longus capitis, rectus capitis anterior, suprahyoid muscles (for chin tuck), infrahyoid muscles (for hyoid stabilization)	Upper cervical flexion	Increase range of motion and modulate abnormal muscle tone	Rhythmic initiation, dynamic reversal, stability reversal, combination of isotonic, contract-relax
Longus colli, platysma, anterior scalene, sternocleidomastoid	Lower cervical flexion	Improve stability	
Contralateral: all scalene muscles; ipsilateral: sternocleidomastoid, longus capitis, longus colli, rectus capitis anterior	Rotation	Enhance strength	
Longus colli, all scalene muscles, sternocleidomastoid	Lateral flexion	Increase range of motion	
Iliocostalis and longissimus capitis, obliquus capitis (superior and inferior), rectus capitis posterior (major and minor), semispinalis capitis and splenius capitis, trapezius	Upper cervical extension	Increase range of motion and modulate abnormal muscle tone	
Iliocostalis cervicis, longissimus cervicis, and splenius cervicis, multifidus and rotatores, semispinalis cervicis, trapezius	Lower cervical extension	Improve stability	
Contralateral: multifidus and rotatores cervicis, semispinalis capitis; ipsilateral: upper trapezius, obliquus capitis inferior, splenius cervicis, splenius capitis	Rotation	Enhance strength	
Iliocostalis cervicis, intertransversarii cervicis, longissimus capitis, obliquus capitis superior, splenius cervicis, splenius capitis, trapezius	Lateral flexion	Increase range of motion	
